# N-of-1 Trials in Healthcare

**DOI:** 10.3390/healthcare9030330

**Published:** 2021-03-15

**Authors:** Suzanne McDonald, Jane Nikles

**Affiliations:** Centre for Clinical Research, The University of Queensland, Building 71/918, Royal Brisbane & Women’s Hospital Campus, Herston, QLD 4029, Australia; catherine.nikles@uq.edu.au

## 1. Introduction

Interest in N-of-1 trials and single-case designs is increasing worldwide, particularly due to the movement towards personalised medicine and patient-centred healthcare. For decades, group-based designs such as the randomised controlled trial have been understood as the “gold standard” for testing treatments, however these designs have provided us with little information about the individual-level improvements in health and well-being outcomes that are of vital importance to healthcare. There is growing recognition of the wide applicability of N-of-1 trials and single-case designs to a number of diverse health disciplines and the value they can bring to clinical research and practice through the focus on understanding individuals.

This Special Issue aimed to showcase novel applications of N-of-1 trials and single-case designs in any health-related discipline, with a specific focus on applications in new health conditions, interventions and contexts, as well as developments in data analysis. This Special Issue presents a collection of thirteen articles that highlight the importance of these methods in both clinical research and practice. Together, the articles report findings from research studies, describe protocols for future studies, and outline key discussion points and opinions for advancing the field. The articles represent a variety of single-case designs, including experimental and observational designs, and demonstrate the substantial flexibility and versatility of N-of-1 trials and single-case studies and their value in healthcare.

## 2. Applications to New Health Conditions and Interventions

The articles in this Special Issue represent a broad application of N-of-1 trials or single-case designs to several health conditions and interventions that have not been previously studied with these designs. For example, Kronish et al. [1] tested bright white light therapy for depressive symptoms in cancer survivors using a series of N-of-1 trials. Participants completed three-weeks of lightbox-delivered bright white light or sham for 30 minutes each morning for 12 weeks and recorded their daily depressive symptoms using a smartphone application that was specially designed for the study. Daza, Wac and Oppezzo [2] used a “self-study” N-of-1 trial design to explore the effects of sleep deprivation on blood glucose, food cravings, and affect in non-diabetic adults. In this study, two of the authors were study participants and provided measurements of activity, sleep, glucose and other outcomes via a continuous glucose meter, fitness tracker, and web-based surveys. Martin, Arden, Porritt, Wildman and Naughton [3] demonstrate the value of applying single-case observational designs to understand temporal relationships. They explored the association between symptoms such as difficulty breathing, tiredness, and pain, and objectively measured nebulizer adherence in people with cystic fibrosis. They used a special nebulizer designed to record information in real time about patient adherence, as well as web-based surveys to record daily symptoms. Two further articles described protocols for future studies; Kaplan et al. [4] plan to use N-of-1 trial designs to evaluate the comparative effectiveness of two diets in paediatric inflammatory bowel disease using a series of N-of-1 trials, and Gimeno and Adlam [5] plan to use single-case experimental designs to evaluate whole-body dynamic seating on activity, participation, and quality of life in dystonic cerebral palsy.

## 3. Contribution to Different Contexts

N-of-1 trials and single-case studies offer a range of opportunities, including testing new treatments to determine individual treatment effects, the ability to compare different doses or combinations of treatments to identify the optimum treatment regime, and for de-prescribing current treatments that may have little or unknown clinical benefit to the patient. In this Special Issue, Clough, Hilmer, Naismith and Gnjidic [6] outline the findings from a pilot study exploring the feasibility of using N-of-1 trials in the context of de-prescribing in older people with dementia. 

N-of-1 trials and single-case designs can play a crucial role in contexts where robust empirical evidence is lacking or insufficient. In this Special Issue, Bradbury, Avila and Grace [7] discuss whether N-of-1 trial designs could become the new “gold standard” approach for evaluating complementary and alternative medicines. They provide an illustrative example of using N-of-1 trials to test probiotics, as an adjunct with regular treatment, for pain associated with fibromyalgia. They provide a convincing argument for the value of N-of-1 trials and single-case designs for the complementary health practitioner. 

Health professionals working in a variety of clinical contexts can benefit from using N-of-1 trials and single-case designs. The individual findings from a study using an N-of-1 trial or single-case design can be used to discuss treatment options with the patient and can lead to shared decision-making regarding the treatment and management of their health condition. Samuel, Holder and Molony [8] have designed a protocol for a systematic review of the literature to identify studies using N-of-1 trials as a clinical tool to support treatment decision-making. The authors plan to comprehensively review the existing evidence to assess the added value of using N-of-1 trials in practice compared to standard care.

## 4. Exploring Statistical Issues

In contrast to case descriptions, N-of-1 trials and single-case designs are scientifically rigorous methods that should be designed a priori and analysed, where possible, using both visual and statistical methods. Many new statistical techniques that address the unique features of individual data collected using N-of-1 trials and single-case designs are emerging in the literature. Several articles in this Special Issue discuss important statistical issues. Wang and Schork [9] used analytical and simulation studies to look at the effect of serial correlation, number of periods/phases, the presence of washout periods, and heteroscedasticity on statistical power in non-randomised AB (or further permutations) alternation designs (where ‘A’ is baseline and ‘B’ is treatment). They showed that the power to detect an effect of the intervention decreased as the strength of serial correlation increased across all the alternation designs they tested. More alternations of ‘AB’ mitigated the decrease in statistical power in the presence of strong serial correlation. The authors also showed that serial correlation reduced when washout periods were used. Tanious and Onghena [10] provide an overview of randomisation tests in different N-of-1 designs. They argue that incorporating an element of randomisation is important for increasing the internal validity and statistical conclusion validity. The article provides a comprehensive illustration of data analysis techniques using randomisation applied to health-related datasets. A series of N-of-1 trials or single-case studies that have used the same protocol can be statistically aggregated to provide conclusions about population-level effects, analogous to a study using a group-based design. In this Special Issue, Blackston, Chapple, McGree, McDonald and Nikles [11] used simulation studies to compare N-of-1 trials to “traditional” designs including parallel and crossover randomised controlled trials. They showed that fewer participants are required in an aggregated N-of-1 study compared to randomised and crossover trials, to obtain the same level of statistical power.

## 5. Challenges Associated with Single-Case Designs

Across the articles in this Special Issue, there are remarks about particular issues and challenges associated with conducting N-of-1 trials. Chalmers, Smeeth and Goldacre [12] describe various issues in regards to implementing N-of-1 trials of statins in the United Kingdom. In particular, they experienced “hyper-regulation” and cultural issues as key barriers to their implementation and uptake in clinical practice rather than scientific, ethical or technical problems. In addition to these barriers, there is often uncertainty about whether N-of-1 trials and single-case studies require ethical approval from an institutional review board, which stems from the debate about whether single-case designs are medical research or clinical care. In this Special Issue, Stunnenberg et al. [13] present a practical flowchart based on an ethical framework aiming to support decision-making about the requirement for ethics approval.

## 6. Summary

Studies using N-of-1 trials and single-case designs are well-suited to complement, strengthen, and generate advances in precision medicine, patient-centred healthcare, and personalised health. We conclude with three important observations for the future of N-of-1 trials and single-case designs based on our reflections on developments in this field and the articles in this Special Issue. First, rapid accelerations in digital technology innovations provide extensive opportunities to gather accurate, unobtrusive real-time health data from individuals —a crucial requirement for N-of-1 trials and single-case studies. Thus, digital technology will play an important role in the future growth of the field of N-of-1 trials and single-case designs. Secondly, single-case designs could contribute substantially to many health disciplines, by providing individual evidence that could then be statistically aggregated to obtain evidence about population-level effects. This approach, which is gaining traction in professions such as complementary and alternative medicine, occupational therapy and dietetics, will optimize therapy for individual patients and will also yield effectiveness data at the group level, because pooling results across similarly conducted N-of-1 trials or single-case studies can generate inferences about the effectiveness of an intervention for a population. This approach could revolutionize the way we conceptualise, study, and determine optimal therapy for individual patients. Finally, there is a need to systematically identify barriers to the implementation and wider adoption of N-of-1 trials and single-case designs. This information can be used to inform strategies to address significant barriers that are preventing these designs from being recognised and accepted as an integral part of clinical research and patient care.

## References

[1] Kronish I.M., Cheung Y.K., Julian J., Parsons F., Lee J., Yoon S., Valdimarsdottir H., Green P., Suls J., Hershman D.L. (2019). Clinical Usefulness of Bright White Light Therapy for Depressive Symptoms in Cancer Survivors: Results from a Series of Personalized [N-of-1] Trials. Healthcare.

[2] Daza E.J., Wac K., Oppezzo M. (2019). Effects of Sleep Deprivation on Blood Glucose, Food Cravings, and Affect in a Non-Diabetic: An N-of-1 Randomized Pilot Study. Healthcare.

[3] Martin R., Arden M., Porritt J., Wildman M., Naughton F. (2020). Investigating the Temporal Relationships between Symptoms and Nebuliser Adherence in People with Cystic Fibrosis: A Series of N-of-1 Observations. Healthcare.

[4] Kaplan H.C., Opipari-Arrigan L., Schmid C.H., Schuler C.L., Saeed S., Braly K.L., Burgis J.C., Nguyen K., Pilley S., Stone J. (2019). Evaluating the Comparative Effectiveness of Two Diets in Pediatric Inflammatory Bowel Disease: A Study Protocol for a Series of N-of-1 Trials. Healthcare.

[5] Gimeno H., Adlam T. (2019). Protocol: Using Single-Case Experimental Design to Evaluate Whole-Body Dynamic Seating on Activity, Participation, and Quality of Life in Dystonic Cerebral Palsy. Healthcare.

[6] Clough A.J., Hilmer S.N., Naismith S.L., Gnjidic D. (2019). The Feasibility of Using N-Of-1 Trials to Investigate Deprescribing in Older Adults with Dementia: A Pilot Study. Healthcare.

[7] Bradbury J., Avila C., Grace S. (2020). Practice-Based Research in Complementary Medicine: Could N-of-1 Trials Become the New Gold Standard?. Healthcare.

[8] Samuel J., Holder T., Molony D. (2019). N-of-1 Trials as a Decision Support Tool in Clinical Practice: A Protocol for a Systematic Literature Review and Narrative Synthesis. Healthcare.

[9] Wang Y., Schork N.J. (2019). Power and Design Issues in Crossover-Based N-Of-1 Clinical Trials with Fixed Data Collection Periods. Healthcare.

[10] Tanious R., Onghena P. (2019). Randomized Single-Case Experimental Designs in Healthcare Research: What, Why, and How?. Healthcare.

[11] Blackston J.W., Chapple A.G., McGree J.M., McDonald S., Nikles J. (2019). Comparison of Aggregated N-of-1 Trials with Parallel and Crossover Randomized Controlled Trials Using Simulation Studies. Healthcare.

[12] Chalmers I., Smeeth L., Goldacre B. (2019). Personalised Medicine Using N-of-1 Trials: Overcoming Barriers to Delivery. Healthcare.

[13] Stunnenberg B.C., Deinum J., Nijenhuis T., Huysmans F., van der Wilt G.J., van Engelen B.G.M., van Agt F. (2020). N-of-1 Trials: Evidence-Based Clinical Care or Medical Research that Requires IRB Approval? A Practical Flowchart Based on an Ethical Framework. Healthcare.

